# Enhancing immunotherapy response in melanoma: myeloid-derived suppressor cells as a therapeutic target

**DOI:** 10.1172/JCI170762

**Published:** 2023-07-03

**Authors:** Feyza Gul Ozbay Kurt, Samantha Lasser, Ihor Arkhypov, Jochen Utikal, Viktor Umansky

**Affiliations:** 1 Skin Cancer Unit, German Cancer Research Center (DKFZ), Heidelberg, Germany.; 2 Department of Dermatology, Venereology and Allergology, University Medical Center Mannheim, Ruprecht-Karls University of Heidelberg, Mannheim, Germany.; 3 DKFZ–Hector Cancer Institute at the University Medical Center Mannheim, Mannheim, Germany.; 4 Mannheim Institute for Innate Immunoscience (MI3), Medical Faculty Mannheim, Ruprecht-Karls University of Heidelberg, Mannheim, Germany.

## Abstract

Despite the remarkable success of immune checkpoint inhibitors (ICIs) in melanoma treatment, resistance to them remains a substantial clinical challenge. Myeloid-derived suppressor cells (MDSCs) represent a heterogeneous population of myeloid cells that can suppress antitumor immune responses mediated by T and natural killer cells and promote tumor growth. They are major contributors to ICI resistance and play a crucial role in creating an immunosuppressive tumor microenvironment. Therefore, targeting MDSCs is considered a promising strategy to improve the therapeutic efficacy of ICIs. This Review describes the mechanism of MDSC-mediated immune suppression, preclinical and clinical studies on MDSC targeting, and potential strategies for inhibiting MDSC functions to improve melanoma immunotherapy.

## Introduction

Melanoma accounts for the vast majority of skin cancer–related deaths. It is known for its high immunogenicity, which makes the disease a suitable target for immunotherapies (1) that deploy the patient’s own immune system to fight against tumors (2). Earlier immunotherapeutic approaches involved the administration of cytokines and interferons, which displayed minimal benefit and considerable toxicities. The application of negative immune checkpoint molecules such as cytotoxic T lymphocyte–associated protein 4 (CTLA-4) and programmed cell death protein 1 (PD-1) as therapeutic targets has revolutionized cancer immunotherapy. Immune checkpoint inhibitors (ICIs) were first administered to patients with advanced melanoma and showed promising results (3). Yet many patients develop diverse resistance mechanisms that decrease the response rate to the treatment (4).

Myeloid-derived suppressor cells (MDSCs) represent a heterogeneous population of myeloid cells with immunosuppressive functions and are known to be enriched in various types of cancer, including melanoma (5–7). The clinical relevance of MDSCs has gained attention owing to the reports showing that increased levels of MDSCs positively correlated with unfavorable clinical outcome and poor survival in different cancer entities (8, 9). Moreover, several reports highlighted a correlation between high MDSC numbers and poor response to ICI. This is attributed to the ability of MDSCs to foster an immunosuppressive tumor microenvironment, which hinders the efficacy of ICIs (10–12).

Current strategies aimed at targeting MDSCs are not entirely effective because of the heterogeneity of these myeloid cells and the complexity of their immunosuppressive function (7). Therapies that included ICIs in combination with MDSC blockers showed potential, yet the absence of definitive markers to identify MDSCs poses a formidable challenge (13). Although several potential MDSC markers and novel factors involved in MDSC accumulation, immunosuppressive functions, and recruitment have been identified, their clinical utility remains to be elucidated.

In this Review, we present current knowledge on the role of MDSCs in the immunotherapy of melanoma. We describe the accumulation, infiltration, and immunosuppressive functions of MDSCs in the tumor microenvironment (TME) as well as existing therapeutic strategies to target them in melanoma-bearing hosts. A special focus is placed on preclinical studies and clinical trials applying MDSC inhibition to overcome resistance to ICIs. Recently identified novel markers for MDSC targeting in melanoma are also discussed.

## Origin and phenotype of MDSCs

MDSCs are immature immunosuppressive myeloid cells or mature myeloid cells that acquired immunosuppressive functions (7, 14). These cells accumulate under chronic inflammatory conditions such as cancer, autoimmune diseases, and chronic infections (14). Under normal conditions, hematopoietic progenitor cells differentiate in the bone marrow into common myeloid progenitors that later give rise to immature myeloid cells, terminally differentiating into macrophages, dendritic cells (DCs), and granulocytes. Pathological conditions such as cancer lead to the dysregulated production of inflammatory signals and hematopoietic growth factors, which can initiate emergency myelopoiesis (15, 16). In this condition, the normal differentiation process of myeloid cells is impaired by the persistent production of growth factors and inflammation signals, resulting in the accumulation of immature myeloid cells (17). In contrast to normal counterparts, these immature myeloid cells, termed MDSCs, display weak phagocytic activity and antiinflammatory and immunosuppressive functions, as well as immature phenotypes and morphologies (18). Further, it has been demonstrated that mature myeloid cells could be converted into MDSCs upon treatment with tumor-derived extracellular vesicles (EVs) (19–21). EVs are abundantly secreted by cancer cells and contain lipids, proteins, RNAs, and microRNAs (miRNAs) (22). It was demonstrated that miRNAs promoted MDSC generation, suppressive function, and expansion via targeting of transcription factors including suppressor of cytokine signaling (SOCS), CCAAT/enhancer binding protein (C/EBP), RUNX family transcription factor 1 (RUNX1), signal transducer and activator of transcription (STAT), and phosphatase and tensin homolog (PTEN) (23). EVs derived from human melanoma cells were shown to skew monocyte differentiation into immunosuppressive cells via a set of miRNAs (miRNA-146a, -155, -125b, -100, -125a, -146b, -99b, and let-7e) (21). It was also found that EVs secreted by mouse or human melanoma cells can convert normal monocytes into immunosuppressive MDSCs via programmed cell death protein ligand 1 (PD-L1) upregulation induced by HSP86/TLR4/NF-κB signaling (20).

MDSCs are categorized into two major subsets, polymorphonuclear (PMN-MDSCs) and monocytic (M-MDSCs), based on their phenotypic and morphological resemblance to granulocytes and monocytes, respectively. In addition, a small subset of human myeloid cells comprising more immature progenitors are defined as early-stage MDSCs (e-MDSCs) (13). In mice, MDSCs are characterized as Gr1^+^ (Ly6G^+^ and Ly6C^+^) CD11b^+^ cells. In mice, PMN-MDSCs are defined as CD11b^+^Ly6G^+^Ly6C^lo^ and M-MDSCs as CD11b^+^Ly6G^−^Ly6C^hi^. In humans, PMN-MDSCs express CD15, CD33, and CD11b, no CD14, and low or no HLA-DR expression, whereas M-MDSCs express CD14, CD33, and CD11b with a lack of CD15 and low or no HLA-DR expression. In addition, e-MDSCs are defined as HLA-DR^–^CD33^+^CD14^–^CD15^–^ cells (18). Human M-MDSCs could be distinguished from monocytes based on the level of HLA-DR expression. In contrast to neutrophils, which are purified on a higher density gradient, PMN-MDSCs are enriched in the low-density fraction after gradient centrifugation using the Ficoll gradient (18).

Additional markers including lectin-like oxidized low-density lipoprotein receptor-1 (LOX-1) have been used to distinguish human PMN-MDSCs from normal neutrophils (24). Moreover, CD84, a member of the signaling lymphocytic activation molecule (SLAM) family, and JAML, a member of the junctional adhesion molecule (JAM) family, have been demonstrated to be coexpressed on human MDSCs and to correlate with MDSC immunosuppressive activity in breast cancer (25). Despite the existence of MDSC markers and their phenotypic definition, the identification of MDSCs is still based on the assessment of their suppressive activity, since they share multiple genes with conventional neutrophils and monocytes (7).

## Accumulation and recruitment of MDSCs

**Figure 1:**
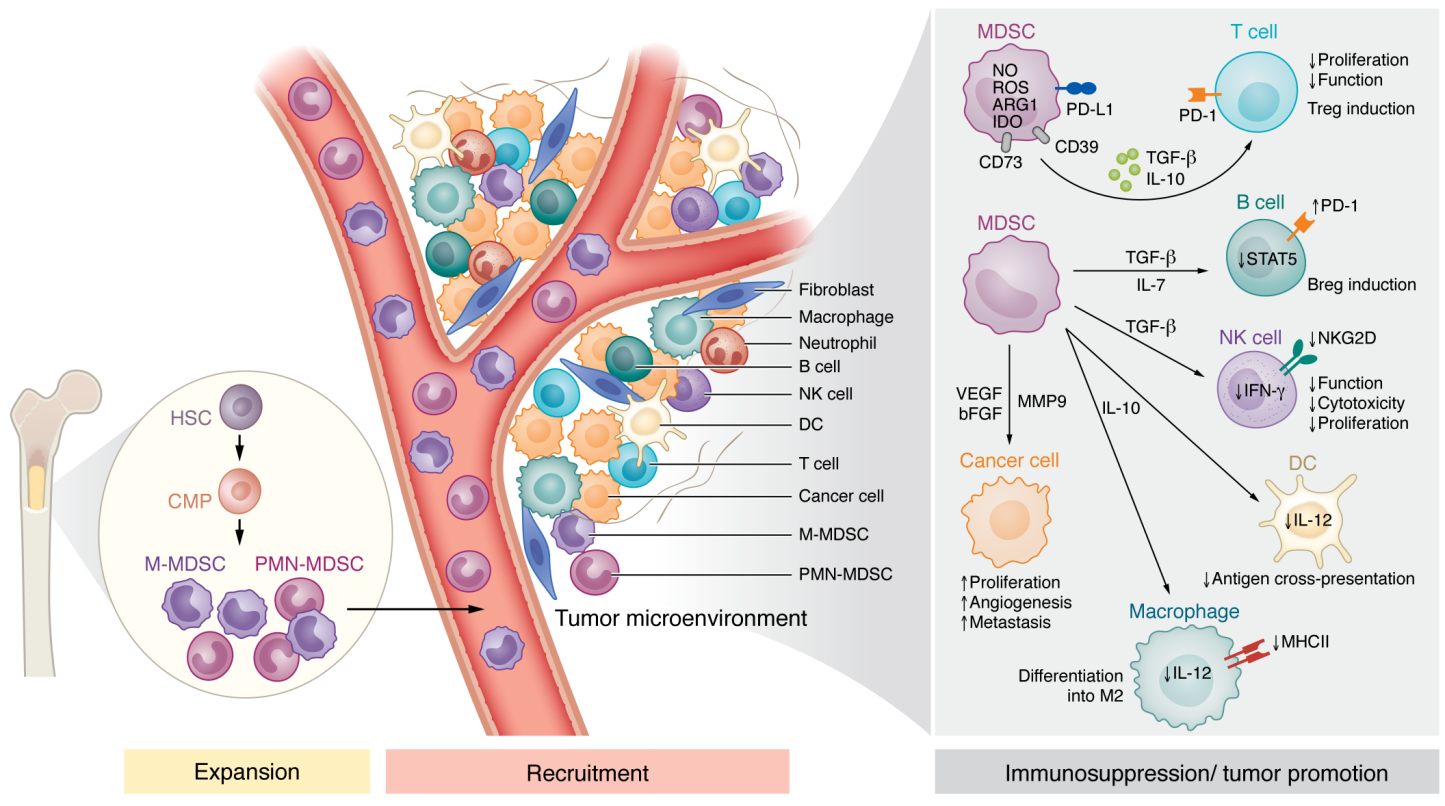
MDSC accumulation, recruitment, and functions in the TME. Inflammatory mediators released by host cells lead to the dysregulation of normal myelopoiesis and to the accumulation of MDSCs in the bone marrow. MDSCs expand and migrate to the TME through the interaction between CCR and CXCR and their corresponding chemokine ligands. In the TME, MDSCs are activated, and promote immunosuppression and tumor growth via various mechanisms. These mechanisms involve inhibiting the functions of T cells, NK cells, and DCs, promoting the differentiation of M2 macrophages, Tregs, and Bregs, and inducing angiogenesis and metastasis. Breg, regulatory B cell; CMP, common myeloid progenitor; HSC, hematopoietic stem cell.

It has been reported that two partially overlapping signals are required for MDSC accumulation and activation (Figure 1) (7). Tumor cell–derived mediators such as stem cell factor (SCF), granulocyte-macrophage colony-stimulating factor (GM-CSF), granulocyte colony-stimulating factor (G-CSF), monocyte colony-stimulating factor (M-CSF), and vascular endothelial growth factor (VEGF) make up the first group of signals that stimulate myelopoiesis and promote MDSC expansion (26). These mediators stimulate STAT and Janus kinase (JAK) proteins. Transcriptional factors or regulators involved in the accumulation of MDSCs include STAT3, STAT4, C/EBP-β, Notch, interferon regulatory factor 8 (IRF8), etc. (27). The second group of signals includes inflammatory cytokines produced mainly by host cells in the TME, such as IL-6, IL-4, IL-1β, and prostaglandin E_2_ (PGE_2_). Other molecules, like Toll-like receptor (TLR) agonists and damage-associated molecular patterns, including high-mobility group box 1 (HMGB1), S100 calcium-binding proteins, tumor-derived heat shock proteins (HSPs), and complement component C5a, are reported to contribute to MDSC generation (14, 28). Long-term secretion of these mediators can promote the activation and immunosuppressive activity of MDSCs. Furthermore, NF-κB, STAT1, and STAT6 transcription factors are also involved in this process (6). Secretion of a high level of cytokines including TGF-β, IL-1β, IL-10, and TNF-α mediates the acquisition of MDSC immunosuppressive features (29). TNF-α was shown to induce the phosphorylation of STAT3, resulting in the differentiation of myeloid progenitor cells into MDSCs (29). Sinha et al. (30) demonstrated that STAT3 induced S100A8/9 proinflammatory proteins, which foster the accumulation of MDSCs. Moreover, inhibition of S100A8/9 has been shown in various mouse tumor models to restrain tumor growth by reducing MDSC accumulation (31–33).

MDSCs are recruited to the TME by chemokines derived from both tumor and stroma cells (Figure 1). It has been demonstrated that chemokines such as CXCL5, CXCL6, CXLC12, CXCL8, CXCL1, CCL2, CCL3, CCL4, and CCL5 play an important role in MDSC recruitment (34). However, these chemokines are also critical for the recruitment of conventional neutrophils and monocytes, indicating the problems associated with specific MDSC targeting (35). Additionally, TME-derived hypoxia is one of the crucial factors stimulating the recruitment of MDSCs (36–38). Hypoxia-inducible factor-1α (HIF-1α) was found to be involved in generating M2 macrophages from monocytes inside a tumor (39).

Several studies indicated that MDSC subsets and tumor entities could determine which chemokines support MDSC migration into the tumor site (40). CCR2 signaling was demonstrated to mediate M-MDSC recruitment, which promoted suppression of CD8^+^ T cell infiltration into the tumor site in melanoma patients (41). The CCR5 ligands CCL3, CCL4, and CCL5 were also reported to play roles in the migration of M-MDSCs (42). On the other hand, PMN-MDSCs are recruited primarily by CXC chemokines such as CXCL1, CXCL2, CXCL5, CXCL6, and CXCL12 produced by tumor cells (43, 44). PMN-MDSCs from melanoma-bearing mice were demonstrated to express CXCR2 (45), and CXCR2 deletion impaired PMN-MDSC accumulation, leading to tumor growth inhibition (45, 46).

## Immunosuppressive functions

MDSCs use several mechanisms to suppress immune responses mediated by T, B, and natural killer (NK) cells, thus strongly accelerating tumor progression. The main mechanisms of T cell suppression are dealing with the expression of negative immune checkpoint molecules like PD-L1, depletion of amino acids required for T cell activation, production of reactive oxygen species (ROS) and nitric oxide (NO), and secretion of TGF-β and IL-10 (Figure 1) (29). MDSCs were shown to reduce the level of several amino acids essential for T cell functions (proliferation and cytokine production), such as cysteine, tryptophan, and l-arginine (47). The upregulation of arginase 1 (ARG1) and inducible NO synthase (iNOS) leads to the depletion of l-arginine amounts in the TME in different cancer entities (48, 49). Another important mechanism involved in MDSC immunosuppressive capacity is the activation of indoleamine 2,3-dioxygenase (IDO), an enzyme that converts l-tryptophan into *N*-formyl kynurenine (50, 51). The deficiency of l-tryptophan results in T cell anergy (51, 52).

A strong production of NO via iNOS activation and ROS by MDSCs was found to promote T cell anergy by the downregulation of T cell receptor ζ chain expression and even induce T cell apoptosis (53–55). Accumulated NO levels were shown to upregulate the expression of cyclooxygenase-2 (COX-2), leading to increased production of PGE_2_ (56). The latter molecule was reported to promote the upregulation of immunosuppressive markers, including ARG1, IDO, and IL-10, in in vitro–generated MDSCs (57, 58).

It has been demonstrated that MDSCs express a high level of PD-L1, which interacts with PD-1 on T cells, inducing their anergy (37). Importantly, tumor-infiltrating MDSCs display higher PD-L1 expression than circulating MDSCs (59). Although some studies demonstrated that PD-L1 expression is predominantly restricted to M- and e-MDSCs (13), other publications described the presence of PD-L1 also on PMN-MDSCs (45, 60). MDSCs can also induce the expansion of regulatory T cells (Tregs) through the secretion of TGF-β and IL-10 (61). Via the expression of the metalloprotease ADAM17, M-MDSCs are able to downregulate the expression of L-selectin (CD62L) on T cells, which impairs their extravasation and tissue infiltration capacity (62). Another mechanism used by MDSCs to suppress T cell function is the production of extracellular adenosine from ATP in the hypoxic TME via ectonucleotidases CD39 and CD73 (63, 64). Adenosine was found to impair the activation of T cells in cancer by inhibiting their proliferation and cytokine production (65).

Besides T cells, MDSCs can inhibit the function of other immune cells, such as NK cells, DCs, macrophages, and B cells. For instance, TGF-β produced by MDSCs was shown to promote the suppression of NK cell functions (66). Crosstalk between MDSCs and NK cells results in impaired NK cell cytotoxicity and induction of immune tolerance by reducing NKG2D expression and IFN-γ production (67). Moreover, PMN-MDSCs were reported to inhibit antigen cross-presentation by DCs in tumor-bearing mice (68). Furthermore, an accumulation of M-MDSCs in melanoma patients blocked DC maturation (6). MDSCs also hinder the function of B cells through the production of IL-7 and STAT5 signaling (69), and upregulate PD-L1 expression on B cells, thereby leading to the accumulation of regulatory B cells (70). Interaction between MDSCs and macrophages leads to the initiation of tumor-promoting immune response through upregulation of IL-10 production by MDSCs and downregulation of IL-12 secretion by macrophages (71).

Metabolic changes in MDSCs have been reported to be associated with the acquisition of their suppressive functions. It has been described that altered lipid metabolism in MDSCs plays a critical role in their differentiation and functions (72). In mice, polyunsaturated fatty acid–enriched diets were found to promote MDSC generation and to enhance MDSC suppressive activity (73). Al-Khami et al. (74) reported that tumor-infiltrating MDSCs increased fatty acid uptake and fatty acid oxidation to foster their immunosuppressive functions and found that intracellular accumulation of lipids in the TME enhanced the oxidative metabolism and activated immunosuppressive mechanisms of MDSCs in a mouse model of Lewis lung carcinoma. It has been recently reported that fatty acid transporter protein 2 (FATP2), responsible for the uptake of arachidonic acid and synthesis of PGE_2_, is involved in the acquisition of PMN-MDSC suppressive activity (75). Inhibition of FATP2 was reported to abrogate PMN-MDSC functions and potentiate the efficacy of cancer immunotherapy in tumor-bearing mice (75). It was also demonstrated that MDSCs exhibit resistance to ferroptosis, a programmed cell death induced by iron-dependent lipid peroxidation (76). In a mouse model of colon cancer, tumor-infiltrating MDSCs were reported to overexpress a key ceramidase, *N*-acylsphingosine amidohydrolase 2 (Asah2), which protects MDSCs from ferroptosis. Correspondingly, in this study, inhibition of Asah2 could reduce MDSC accumulation in colon tumors (77).

Furthermore, MDSCs can upregulate glycolytic pathways, which support their survival by preventing ROS-mediated apoptosis (78). M-MDSCs isolated from tumor tissue of patients with hepatocellular carcinoma displayed reduced cellular ATP content and failed to utilize glucose, which is mediated by the accumulation of methylglyoxal in MDSCs. By transferring methylglyoxal to T cells, MDSCs can suppress their function owing to the depletion of cytosolic amino acids such as l-arginine (79).

The TME is characterized by hypoxia, nutrient deprivation, acidic pH, and elevated levels of free radicals, which could stimulate the activation of ER stress sensors such as inositol-requiring enzyme-1 (IRE1), protein kinase RNA-like endoplasmic reticulum kinase (PERK), and activating transcription factor 6 (ATF6) that leads to the induction of ER stress in MDSCs (80). As a response to ER stress, the expression of C/EBP homologous protein (CHOP) is enhanced, resulting in the activation of proapoptotic genes. Enhanced CHOP expression in both human and mouse MDSCs contributed to their short lifespan and correlated with the ability of MDSCs to impair T cell responses (81).

In addition to their immunosuppressive function, MDSCs were shown to regulate tumor angiogenesis and vasculogenesis by producing high levels of matrix metalloproteinase 9 (MMP9) (82). Through STAT3 activation, MDSCs can also directly produce angiogenic factors like VEGF and basic fibroblast growth factor (bFGF) (83).

## MDSCs in melanoma

Melanoma originates from the malignant transformation of melanocytes that can be found in different anatomic sites, including skin, conjunctiva, mucosal surfaces, and uveal structures (1, 84). Malignant melanoma is considered to be the most aggressive and fatal form of skin cancer. It results predominantly from oncogenic drivers, leading to constitutive activation of the MAPK pathway, including mutations of BRAF (40%–50% of cases), NRAS (20%–30% of cases), and NF1 (10%–15% of cases) (85). Selective inhibitors of mutant BRAF (dabrafenib, encorafenib, or vemurafenib) are currently used in combination with MEK inhibitors (trametinib, binimetinib, or cobimetinib) for the treatment of metastatic melanoma (85). Compared with the earlier chemotherapeutic approaches, lower toxicity and increased overall survival have been achieved with BRAF inhibitor treatment (86). However, the treatment efficiency of MEK and BRAF inhibitors remains low since a significant number of patients acquire resistance (87). Studies reported that BRAF and MEK inhibitors could exert immunomodulatory effects (88). BRAF inhibition was reported to reduce the recruitment of MDSCs in the TME in melanoma-bearing mice (89). Furthermore, MEK inhibition was shown to prevent the polarization of monocytes into MDSCs and the infiltration of MDSCs into the TME in a mouse melanoma model (90). On the other hand, Steinberg et al. (91) reported that in mice with melanoma resistant to BRAF inhibitors, MDSC functions were restored after initial reduction, indicating the potential role of MDSCs in the acquisition of such resistance.

Melanoma has a high mutational burden and shows high immune infiltration, which makes it an ideal target for immunotherapy (92). Currently, approved immunotherapeutic regimens include nivolumab and pembrolizumab to target PD-1, ipilimumab to target CTLA-4, or a combination of antibodies against PD-1 and CTLA-4 (93). Administration of ICIs displayed initially a high response rate and improved clinical outcomes (94, 95). However, many patients develop various resistance mechanisms, which reduce the response rate to the treatment (4, 96). Resistance to ICIs primarily resulted in insufficient generation or dysfunction of antitumor effector T cells or inadequate formation of memory T cells (97). Since the TME was shown to determine the effectiveness of ICIs, tumor-infiltrating immune cells represent promising targets to improve the effect of ICIs (98). Melanoma cells produce various factors that induce the generation and enrichment of MDSCs, Tregs, cancer-associated fibroblasts, and tumor-associated macrophages (99, 100). Among these immunosuppressive cell populations, MDSCs are considered to play a major role in the immunosuppressive melanoma microenvironment (98, 99).

Chronic inflammation was reported to be associated with melanoma initiation and progression (101). Melanoma cells are able to produce various inflammatory mediators, such as GM-CSF, VEGF, TGF-β, TNF-α, IL-6, IL-1β, IL-10, and chemokines (CCL2, CCL5, CXCL1, CXCL2, CXCL8, CXCL10). They can also induce the production of cytokines, chemokines, and growth factors by fibroblasts or immune cells, which can further stimulate the chemokine production by tumor cells, thereby creating autocrine and paracrine loops important for tumor progression (102). Long-term secretion of such inflammatory mediators induced MDSC accumulation and activation as well as the conversion of normal myeloid cells (like monocytes) into immunosuppressive MDSCs (102, 103).

Studies with *RET*-transgenic mice, which are characterized by the spontaneous development of skin malignant melanoma, revealed a profound accumulation of several inflammatory factors in melanoma lesions associated with MDSC enrichment in the melanoma microenvironment (104). These MDSCs strongly expressed ARG1 and PD-L1, produced high amounts of NOS and ROS, and significantly inhibited T cell functions both in vitro and in vivo (104). Similar observations have been demonstrated in the peripheral blood of melanoma patients. Elevated numbers of M-MDSCs in advanced melanoma patients were found to be associated with a high level of inflammatory mediators such as IL-1β and IFN-γ, which promotes MDSC accumulation and activation (105, 106). Other reports also showed an association between high levels of peripheral M-MDSCs and PMN-MDSCs and the tumor burden in patients with malignant melanoma (107, 108).

Several lines of evidence have illustrated the role of miRNAs in the expansion and activation of MDSCs (23). Huber et al. (21) reported that elevated expression of a set of miRNAs was significantly correlated with shorter progression-free survival in patients undergoing treatment with ipilimumab and nivolumab. Moreover, cancer stem cells could recruit MDSCs to regulate immunosuppression in the TME. For instance, downregulation of miRNA-92 expression in CD133^+^ melanoma stem cells potentiated the accumulation of MDSCs in the tumor site by enhancing integrin-dependent activation of TGF-β in melanoma-bearing mice (109).

## Targeting MDSCs in melanoma therapy

**Figure 2:**
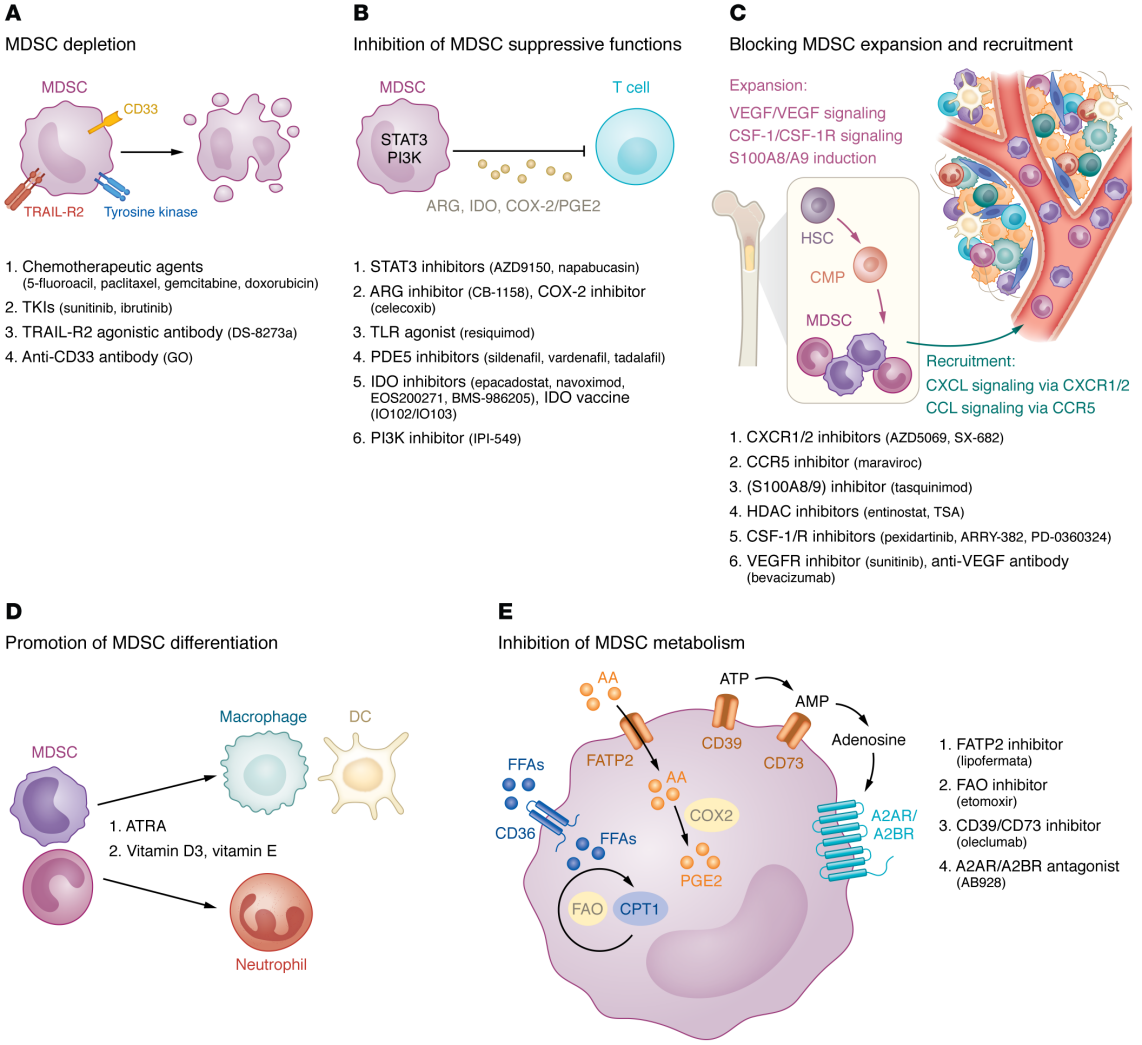
Potential therapeutic approaches to target MDSCs. The main strategies to target MDSCs include MDSC depletion (**A**); inhibition of MDSC suppressive functions (**B**); blocking of MDSC expansion and recruitment (**C**); promotion of MDSC differentiation (**D**); and inhibition of MDSC metabolism (**E**). Relevant MDSC mechanisms are illustrated in each panel, and examples for each type of therapeutic approach are listed. AA, arachidonic acid; CMP, common myeloid progenitor; CPT1, carnitine palmitoyltransferase 1; FAO, fatty acid oxidation; FFAs, free fatty acids; GO, gemtuzumab ozogamicin; HSC, hematopoietic stem cell; TKIs, tyrosine kinase inhibitors; TSA, trichostatin A.

A number of preclinical and clinical studies have been performed to evaluate the efficacy and safety of MDSC inhibition either as a single treatment or in combination with other therapies to improve antitumor responses and overcome the resistance of cancer cells (110–115). Ongoing clinical trials targeting MDSCs in melanoma patients are listed in Table 1. Current treatment strategies can be classified into five groups: (a) depletion of MDSCs; (b) inhibition of their suppressive functions; (c) blocking of their expansion and recruitment to the tumor site; (d) promotion of MDSC differentiation into mature myeloid cells; and (e) inhibition of MDSC metabolism (Figure 2) (35, 116).

### MDSC depletion.

Such chemotherapeutics as gemcitabine, 5-fluorouracil, paclitaxel, and doxorubicin were demonstrated to significantly reduce MDSC frequencies (116). Thus, low-dose paclitaxel could decrease the accumulation and immunosuppressive activity of tumor-infiltrating MDSCs in melanoma-bearing mice (117) and melanoma patients (118), leading to the inhibition of tumor progression. Moreover, the anti-CD33 monoclonal antibody gemtuzumab ozogamicin has recently been reported to deplete MDSCs, restore T cell immunity, and improve the efficiency of immunotherapy of various tumors, including melanoma (119). Furthermore, the tyrosine kinase inhibitor sunitinib, which blocks several tyrosine kinases localized on both MDSCs and tumor cells, was reported to reduce MDSC frequencies via blockade of Fms-like tyrosine kinase 3 (Flt3), c-kit (CD117), and VEGF receptor (VEGFR) in patients with renal cell carcinoma (120, 121). Additionally, the TNF-related apoptosis-induced ligand receptor 2 (TRAIL-R2) agonistic antibody DS-8273a was demonstrated to eliminate MDSCs without affecting mature myeloid cells and to diminish the progression of disease among a cohort of patients with advanced malignancies (96). A phase I trial also tested the efficacy and safety of DS-8273a in combination with nivolumab in unresectable stage III/IV melanoma patients (ClinicalTrials.gov NCT02983006).

### Inhibition of MDSC suppressive functions.

Disruption of COX-2/PGE_2_ pathway and phosphodiesterase-5 (PDE5) inhibitors such as sildenafil, vardenafil, and tadalafil has been employed to neutralize MDSC immunosuppressive capacities (35). Sildenafil was reported to reduce the expression of ARG1 and iNOS in MDSCs, and thereby inhibit their immunosuppressive functions (122). Furthermore, Meyer et al. (104) showed that sildenafil prolonged the survival of melanoma-bearing mice by reducing MDSC levels and activity, leading to restored CD8^+^ T cell infiltration and function in the TME. In an open-label trial with tadalafil, some metastatic melanoma patients resistant to ICI showed a response to the treatment that was associated with MDSC inhibition and accumulation of activated CD8^+^ T cells in metastatic lesions (123).

Blocking phosphatidylinositol 3-kinase (PI3K) was reported to reprogram MDSCs from an immunosuppressive to an immune-promoting phenotype (82). An ongoing phase I clinical trial with IPI-549, an inhibitor of PI3K, in combination with nivolumab is demonstrating improved clinical activity and safety in patients with stage III/IV melanoma who showed resistance to anti–PD-L1 therapy (NCT02637531).

Inhibiting IDO could be another strategy to block MDSC functions. Clinical trials in patients with advanced solid tumors using IDO inhibitors such as epacadostat (124), navoximod (NCT02048709), EOS200271 (125), and BMS-986205 (NCT02658890) in combination therapies with ICIs showed that the treatment was effective and well tolerated. However, in a phase III trial, the combination of epacadostat with pembrolizumab in patients with unresectable or metastatic melanoma was not successful (NCT02752074). A preclinical study using an IDO vaccine to target IDO^+^ immunosuppressive cells in the TME demonstrated a depletion of immunosuppressive myeloid populations and improvement in antitumor effects in both IDO-expressing and non-IDO-expressing tumors from melanoma-bearing mice (114). Moreover, a phase I/II clinical trial in metastatic melanoma patients including an immunomodulatory vaccine (IO102/IO103) against IDO and PD-L1 showed a high response rate and improved progression-free survival (112).

STAT3 was found to be a promising target to diminish MDSC immunosuppressive functions (126). Diverse approaches targeting STAT3 inhibition have been evaluated in preclinical models and clinical trials (127–129). Nevertheless, clinical implementation in advanced solid tumors has yielded limited efficacy or intolerable toxicities (130). We demonstrated previously that STAT3 inhibition by napabucasin reduced the immunosuppressive activity of MDSCs and prolonged the survival of melanoma-bearing mice (131). Moreover, STAT3 activation in circulating M-MDSCs from melanoma patients was found to be correlated with their poor progression-free survival, indicating the potential role of STAT3 as a predictive marker and a therapeutic target in melanoma (131).

The key cytokine IL-1β, produced by inflammasome in response to damage-associated or pathogen-associated molecular patterns, was reported to be enriched in melanoma patients (132). Tengesdal et al. (133) demonstrated that inhibition of tumor-derived NLR family pyrin domain containing 3 (NLRP3) inflammasome by dapansutrile (OLT1177) in combination with ICIs reduced MDSC-mediated T cell suppression and thereby decreased tumor progression in melanoma-bearing mice.

### Blocking MDSC expansion and recruitment.

Growth factors such as SCF, GM-CSF, CSF, and VEGF are produced by tumor cells and could stimulate the expansion of MDSCs (134–136). Inhibiting MDSC development from the bone marrow progenitors by blocking SCF was reported to reduce MDSC expansion and tumor angiogenesis in a mouse model of colon cancer (137). Moreover, the blockade of GM-CSF/G-CSF signaling was reported to restrain the accumulation of MDSCs and reinvigorate antitumor immune responses (138). Furthermore, when combined with other therapies, CSF-1/CSF-1R blockade was also shown to inhibit MDSC expansion (139). A clinical trial with the CSF-1R inhibitor ARRY-382 in patients with advanced solid tumors including melanoma was terminated due to insufficient efficacy (NCT02880371). However, a phase I/II clinical trial to test the efficacy and safety of CSF-1R inhibitors (PD-0360324) in patients with melanoma is still ongoing (NCT02554812).

Blocking the interactions of chemokine receptors with their ligands to inhibit MDSC recruitment to the tumor site has been implicated as a therapeutic strategy. Anti-CXCR2 therapy was shown to reduce the accumulation of PMN-MDSCs in the TME, prolong survival, and decrease the occurrence of distant metastases in melanoma-bearing mice (45). Currently, SX-682, a CXCR1/2 inhibitor, is being tested in a phase I trial with pembrolizumab in patients with metastatic melanoma (NCT03161431).

Histone deacetylases (HDACs) and DNA methyltransferases can regulate antitumor immunity (140). HDAC inhibition has been shown to reduce MDSC recruitment to the tumor site, reinforce T cell activation, and thereby improve antitumor immune responses (141). The HDAC inhibitor entinostat applied in patients with metastatic uveal melanoma in combination with pembrolizumab was reported to promote durable antitumor responses (142). Moreover, Li et al. (143) reported that low-dose HDAC inhibitor trichostatin A in combination with anti–PD-L1 antibodies potentiated antitumor effects of immunotherapies and prolonged the survival of melanoma-bearing mice.

A number of reports demonstrated that MDSCs contribute to tumor growth by stimulating angiogenesis (83, 144, 145). In particular, MDSCs increase the proliferation and vasculogenic mimicry formation of melanoma cells (146). It has been demonstrated that the chemotherapeutic drug doxycycline remarkably reduced the ability of MDSCs to stimulate mimicry formation in melanoma cells, resulting in a strong antitumor effect when applied in combination with anti–PD-1 antibodies in melanoma-bearing mice (146). Moreover, anti-VEGF/VEGFR agents tested in clinical trials could reduce the recruitment of MDSCs and inhibit their angiogenesis-promoting effects in patients with metastatic non–small cell lung cancer (NSCLC) and colorectal cancer (147, 148). Additionally, a receptor for the proangiogenic factor angiopoietin 2, TIE-2, was reported to be expressed on circulating M-MDSCs from melanoma patients (149). TIE-2^+^ M-MDSCs overexpressed PD-L1, CD73, IL-10, and TGF-β and displayed high immunosuppressive activity against melanoma-specific T cells. The authors suggested that NGPT2/TIE-2 signaling represents a tumor escape mechanism and that the combination of TIE-2 inhibitors and ICIs possesses therapeutic potential in melanoma (149).

Sun et al. (150) reported that the level of CXCL10 is greatly enhanced under tumor conditions and increased CXCL10 induces the accumulation of peripheral M-MDSCs, ultimately leading to tumor growth and metastasis in melanoma-bearing mice. This may indicate the importance of potential therapies targeting CXCL10 in the TME.

Artemisinin, an antimalarial drug, has been described as a promising therapeutic agent for cancer treatment (151). A preclinical study demonstrated that artemisinin therapy inhibited the accumulation and immunosuppressive activity of MDSCs, promoted antitumor T cell proliferation, and enhanced the efficacy of anti–PD-L1 therapy in melanoma-bearing mice (152).

### Promotion of MDSC differentiation.

All-*trans* retinoic acid (ATRA) was demonstrated to stimulate the maturation of myeloid cells into fully differentiated and less immunosuppressive variants (153). ATRA-induced differentiation of MDSCs into mature myeloid cells has been implicated in many preclinical and clinical studies (153, 154). In particular, a phase I/II clinical trial with a combination of ATRA and pembrolizumab revealed a favorable tolerability and high response rate in patients with stage IV melanoma (NCT02403778) (115).

### Inhibition of MDSC metabolism.

Another possibility to inhibit MDSC-mediated immunosuppression is to interrupt MDSC metabolism. In several tumor mouse models, the FATP2 inhibitor lipofermata alone or in combination with ICI blocked the activity of PMN-MDSCs and delayed tumor growth (75). Etomoxir is a small-molecule inhibitor of fatty acid oxidation (FAO), blocking carnitine palmitoyltransferase 1a (CPT1a), an important transporter critical for the oxidation of long-chain fatty acids in mitochondria (155). FAO inhibition by etomoxir was reported to decrease tumor growth in different mouse models by limiting MDSC fatty acid metabolism (156). Several studies demonstrated the association between the CD39/CD73/A2AR signaling pathway and poor cancer prognosis (64, 157, 158). An anti-CD73 antibody, oleclumab, is being tested together with anti–PD-L1 antibody (durvalumab) in various phase II trials in patients with NSCLC (NCT03822351, NCT03334617, NCT03794544). Moreover, clinical studies have also been performed to evaluate the safety and efficacy of the dual inhibition of adenosine receptors A2AR and A2BR (NCT05024097) or coinhibition of A2AR and CD73 (159) as a potential strategy to inhibit adenosine-mediated immunosuppression.

Itaconate, a tricarboxylic acid cycle–derived metabolite produced after the activation of immune response gene 1 (IRG1) by inflammatory stimuli, was found to be secreted by MDSCs (113, 160). Zhao et al. (160) demonstrated that itaconate derived from MDSCs suppressed CD8^+^ T cell proliferation and function. Furthermore, a loss of IRG1 diminished tumor growth and potentiated the efficacy of anti–PD-1 immunotherapy in the murine melanoma model, suggesting that IRG1 could be targeted to improve response to ICIs.

## Novel markers for MDSC targeting in melanoma

Among potential novel surface markers for MDSC identification, another lipid transport receptor, CD36, was found to be upregulated in tumor-infiltrating PMN-MDSCs with increased immunosuppressive functions in patients with renal cell carcinoma and colon adenocarcinoma (74). The deletion of CD36 or FAO inhibition resulted in decreased suppressive functions of MDSCs and increased efficacy of immunotherapy associated with delayed tumor growth in a mouse model of Lewis lung carcinoma (74).

General control nonderepressible 2 (GCN2), a serine-threonine kinase controlling transcription and translation in response to nutrient availability (161), was found to be a critical driver for the generation of tumor-associated macrophages and MDSCs in tumor-bearing hosts (162). Depletion of GCN2 decreased MDSC immunosuppressive functions and reduced IFN-γ expression in intratumoral CD8^+^ T cells in a mouse melanoma model (162). Increased GCN2 activity was also found to be correlated with a decreased overall survival in melanoma patients (162).

Another potential MDSC marker, immunoglobulin-like transcript 3 (ILT3), was found to play an important role in the acquisition of their immunosuppressive activity (163). MDSCs generated via melanoma cell lines were found to express high levels of ILT3, and ILT3 inhibition reduced the capacity of MDSCs to suppress T cells (164). Moreover, the ILT3^hi^ fraction of PMN-MDSCs were shown to be correlated with poor outcome in NSCLC patients (165), indicating that blocking of ILT3 may increase the antitumor responses by inhibiting MDSC functions.

## Conclusion and future perspectives

ICI therapies have led to a significant improvement in the treatment of metastatic melanoma over the past decade. However, this improvement is not sufficient, and resistance develops over time due to a profound immunosuppression in the TME. MDSCs are strongly enriched in the melanoma microenvironment and employ several mechanisms to inhibit antitumor effector functions of T and NK cells (103). To enhance cancer immunotherapy, it is critically important to reprogram the unresponsive T cells in the TME to reinvigorate their antitumor functions (2). In this context, we have summarized the current understanding of the mechanisms by which MDSCs could support the immunosuppressive melanoma microenvironment, as well as the current strategies that aim at MDSC inhibition to enhance antitumor immune responses and overcome the resistance to immunotherapy. A thorough understanding of the complex interactions between MDSCs and tumor cells as well as with other immune cells and the identification of novel surface markers that can distinguish MDSCs from normal myeloid cells are needed to develop more effective melanoma immunotherapies. However, some challenges exist in MDSC targeting. Blocking one single mechanism might not be sufficient to inhibit MDSC functions, since these cells are able to apply various suppression mechanisms simultaneously. Therefore, new studies are needed to find factors that could target more than one mechanism of suppression. Moreover, since MDSCs share multiple phenotypic similarities with their normal counterparts, many MDSC-targeting compounds suffer from a lack of specificity. Refinement of phenotypic definition is needed to modulate MDSCs without causing an impact on conventional monocytes and neutrophils. The use of high-dimensional single-cell technologies could be crucial for reducing the ambiguity by creating a precise definition of MDSCs, enabling functional manipulation, and ultimately incorporating MDSC targeting into clinical practice.

## Figures and Tables

**Table 1:**
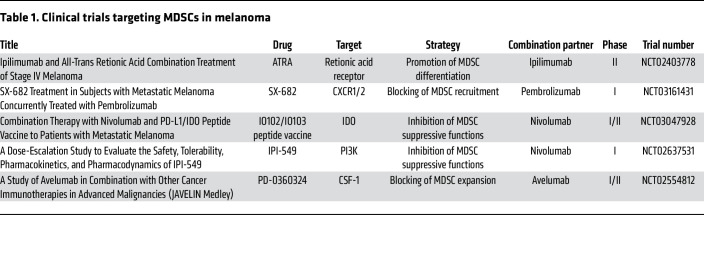
Clinical trials targeting MDSCs in melanoma

## Version 1 07/03/2023

Electronic publication
